# Genetic and Metabolic Markers for Cognition: The Interplay of Hyperhomocysteinemia and ApoE Genotype

**DOI:** 10.1002/alz70856_106720

**Published:** 2026-01-07

**Authors:** Sandhya Gandhi Natarajan, Monisha S, Thomas Gregor Issac

**Affiliations:** ^1^ Centre for Brain Research, Bangalore, karnataka, India; ^2^ Centre for Brain Research, Indian Institute of Science, Bengaluru, Karnataka, India; ^3^ Centre for Brain Research, Indian Institute of Science, Bangalore, Karnataka, India

## Abstract

**Background:**

The presence of ApoE4 allele is known to be the most important genetic risk factor for Late Onset Alzheimer's Disease (LOAD). Hyperhomocysteinemia (HHcy), a cardiovascular risk factor, is also being extensively studied for its role in increasing the risk of cognitive decline in the elderly. The current study aims to explore if any interaction exists between HHcy and ApoE4 allele with respect to their association with cognition.

**Method:**

The study recruited 750 participants from the Tata Longitudinal Study of Ageing (TLSA), an ongoing cohort study of individuals aged ≥ 45 years. The Computerised Assessment of Adult Information Processing (COGNITO) neuropsychological battery was used to assess cognitive performance. Homocysteine levels were quantified using chemiluminescence immunoassays. ApoE genotype was determined using Polymerase Chain Reaction (PCR) amplification followed by Sanger sequencing. Generalised Linear Model (GLM) was used to study the interaction between HHcy and ApoE4 allele on cognition.

**Result:**

The GLM identified that the HHcy/ApoE4 non‐carriers had impaired performance in reading and sentence comprehension (B=‐0.190; *p* = 0.047) and form matching (B=‐0.335; *p* = 0.029) tasks. Such impairment was not observed in the HHcy/ApoE4 carriers group.

In the name‐face association task, only the HHcy/ApoE E4 carriers group (B=‐0.672; *p* = 0.037) had lower scores when compared to the reference group. In episodic memory immediate recall task, both HHcy/ApoE E4 non‐carriers (B=‐0.323; *p* = 0.015) and HHcy/ApoE E4 carriers (B=‐0.423; *p* = 0.040) groups had lower scores.

**Conclusion:**

The results suggest that the association of HHcy with impairment in memory‐related tasks was seen irrespective of ApoE4 carrier status, but is exaggerated in ApoE4 carriers. In tasks related to language and visuo‐spatial abilities, the ApoE4 carriers had no association with the scores even if they had HHcy. Thus, the study results imply that HHcy affects cognitive functioning, but ApoE4 allele modulates the relationship only in non‐memory domains.

